# Geochemical Assessment and Spatial Analysis of Heavy Metals in the Surface Sediments in the Eastern Beibu Gulf: A Reflection on the Industrial Development of the South China Coast

**DOI:** 10.3390/ijerph15030496

**Published:** 2018-03-12

**Authors:** Fajin Chen, Jing Lin, Bihua Qian, Zhai Wu, Peng Huang, Kai Chen, Tianyao Li, Minggang Cai

**Affiliations:** 1 Guangdong Province Key Laboratory for Coastal Ocean Variation and Disaster Prediction, Guangdong Ocean University, Zhanjiang 524088, China; fjchen04@163.com; 2 College of Ocean and Meteorology, Guangdong Ocean University, Zhanjiang 524088, China; penghuang@xmu.edu.cn; 3 Third Institution of Oceanography, State Oceanic Administration, Xiamen 361005, China; linjing@tio.org.cn; 4 College of Ocean and Earth Sciences, Xiamen University, Xiamen 361102, China; qbh40@126.com (B.Q.); 22320151152094@stu.xmu.edu.cn (Z.W.); 22320142200993@stu.xmu.edu.cn (T.L.); 5 Marine Environment and Fishery Monitoring Station of Quanzhou, Quanzhou 362000, China; 6 Coastal and Ocean Management Institute, Xiamen University, Xiamen 361102, China; kaychen@stu.xmu.edu.cn

**Keywords:** heavy metals, assessment, distribution, surface sediments, eastern Beibu Gulf

## Abstract

The Beibu Gulf (also named the Gulf of Tonkin), located in the northwest of the South China Sea, is representative of a bay suffering from turbulence and contamination associated with rapid industrialization and urbanization. In this study, we aim to provide the novel baseline levels of heavy metals for the research area. Concentrations of five heavy metals (i.e., Cu, Pb, Zn, Cd and Cr) were determined in surface sediments from 35 sites in the eastern Beibu Gulf. The heavy metal content varied from 6.72 to 25.95 mg/kg for Cu, 16.99 to 57.98 mg/kg for Pb, 73.15 to 112.25 mg/kg for Zn, 0.03 to 0.12 mg/kg for Cd, and 20.69 to 56.47 mg/kg for Cr, respectively. With respect to the Chinese sediment quality criteria, sediments in the eastern Beibu Gulf have not been significantly affected by coastal metal pollutions. The results deduced from the geoaccumulation index (I_geo_) showed that the study area has been slightly polluted by Pb, which might be caused by non-point sources. Relatively high concentrations of Cu, Pb and Cd were found around the coastal areas of Guangxi province, the Leizhou Peninsula and the northwest coast of Hainan Island, whereas the highest concentrations of Zn and Cr were found on the northwest coast of Hainan Island. Spatial distribution patterns of the heavy metals showed that bioavailable fractions of Pb were higher than in the residual fractions, while Cu and Cd concentrations in exchangeable and carbonate fractions were relatively higher than those in the bioavailable fractions. Hierarchical clustering analysis suggested that the sampling stations could be separated into three groups with different geographical distributions. Accompanying their similar spatial distribution in the study area, significant correlation coefficients among Cu, Cd and Pb were also found, indicating that these three metals might have had similar sources. Overall, the results indicated that the distribution of these heavy metals in the surface sediments collected from the Beibu Gulf was complex.

## 1. Introduction

The Beibu Gulf (i.e., Gulf of Tonkin), located in the northwest of the South China Sea (SCS), is representative of a tropical sea regime that is significantly affected by the monsoon. Beibu Gulf is a very important area for the economic progress of nearby countries (e.g., China and Vietnam), and it is considered as a traditional fishing ground because of its high productivity and rich biological diversity. As the largest area of extant mangrove and sea grass in China, the Beibu Gulf is also a main source of fishery products for coastal cities, such as clam, crabs and varieties of fishes [1,2,3,4]. Therefore, it is a significant fishery and has also become the largest free trade area in South China [5,6]. However, during recent decades there has been high speed economic development and urban construction in the cities surrounding the eastern Beibu Gulf. The Beibu Gulf Economic Zone, which was established in 2008, has promoted economic activity. For example, total production increased at a rate of 15.6% in 2010 [7]. Meanwhile, industries such as equipment production, petroleum processing and metal smelting—specifically Qinzhou’s oil refining projects and Fangcheng Port’s metal industries—have been developing rapidly [7]. The development of the Beibu Gulf Economic Zone results in the emission of large amounts of discharged materials into the Beibu Gulf via river input and atmospheric deposition. Heavy metals are usually treated as one of the most serious pollutants due to their potentially toxic effects, environmental persistence and bioaccumulation capacity [8,9,10,11]. Heavy metal pollution in aquatic environments has received much attention in China in the past decade [6,12,13,14,15,16]. With economic development and urbanization, the estuaries and coastal ecosystems in China are now facing increasingly heavy metal pollution stress. It was reported that 29,720 km^2^ of offshore areas of China were heavily polluted, and about 30,000 t of metals were discharged into the coastal environment by major rivers every year between 2002 and 2008 [17].

Heavy metals that have natural and anthropogenic sources are introduced into coastal areas and thereby possibly impact marine organisms by several pathways, including river runoff, atmospheric deposition, and industrial and agricultural activities [18,19,20]. There are some rivers in the south of Guangxi, the southwest of Guangdong, and western Hainan Island, that carry industrial, agricultural, and urban domestic wastewater into the eastern Beibu Gulf. In addition, seawater in the eastern Beibu Gulf may also be influenced by discharge from the Pearl River that runs through the Qiongzhou Strait [21]. Metals in aquatic ecosystems can also be adsorbed on suspended particles which then settle onto surface sediments [22]. That is to say, sediments are regarded as both sinks and potential secondary sources of heavy metals, and play an important role in the geochemical behavior of heavy metals [23,24].

Although there have been reports about some heavy metals in the local region of the Beibu Gulf, most of the focus has been on the near-shore sites and some targeted regions around oil platforms [25]. There were seldom results indicating heavy metals in the sediments for the areas that were relatively open sea. These studies were mainly conducted since 2010, and focused on the distribution of metals, background values, and historical records. Only one study reports on metals in sediments for the entire eastern Beibu Gulf. Our study could fill the gap in providing data on the heavy metal content of marine sediments in a marginal sea of China. The objectives of this work were to determine and evaluate the heavy metals content in the surface sediments from the eastern Beibu Gulf, secondly, to discuss their distribution patterns and possible sources, and thirdly to assess the sedimentological factors that affect their distribution. 

## 2. Materials and Methods

### 2.1. Study Area and Sample Collection

**Figure 1:**
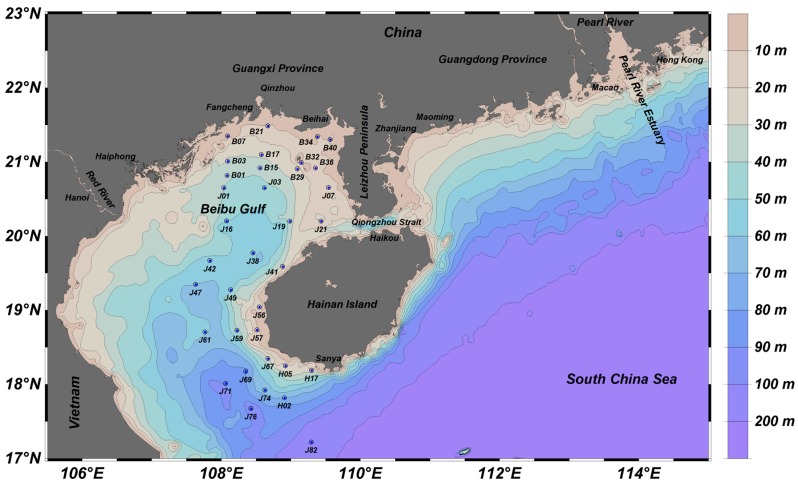
Sampling stations for surface sediments in the Beibu Gulf.

The Beibu Gulf (17.0~22.0° N, 105.5~110.0° E) is a semi-enclosed gulf located in the northwest of the South China Sea (SCS). The depth of the Gulf ranges from 10 to 60 m, with a mean value of 38 m. It has an area of approximately 128,000 km^2^ [26], and connects to the SCS through a narrow ocean channel (the Qiongzhou Strait) to the east and between Hainan Island and the Vietnamese coast to the south (Figure 1).

Marine surface sediment samples were collected from 35 stations in the eastern Beibu Gulf in October 2007 (Figure 1). These stations have good coverage of the study area. Surface sediment samples (to a depth of 3 cm) were taken with a grab sampler. Sediment samples were removed from the sampler using a plastic spoon, then placed into acid-washed plastic bags and stored at −4 °C before processing and analyzing.

### 2.2. Sample Pretreatment and Analysis

Sediment samples were freeze-dried for at least 24 h and then ground to pass through a 160 mesh sieve using an agate mortar and pestle. For metal analysis, samples were digested with the mixture of HNO_3_ and HClO_4_ as described by Cai [27]. Samples were then examined for Cu, Pb, Zn, Cd and Cr concentrations using an atomic absorption spectrophotometer (SOLAAR M6, Thermo Fisher Scientific, Waltham, MA, USA). Redox potential (Eh) was recorded in situ using a potentiometer. Granulometry was determined using a Mastersizer 2000 laser particle size analyzer. Organic carbon was measured based on potassium dichromate oxidation–ferrous sulphate titrimetry and sulfide was measured by iodimetry.

### 2.3. Quality Control

Ultrapure water was used throughout the study. All glassware was previously soaked in nitric acid (1:3) for at least 7 days and then rinsed with ultrapure water. Method blanks were prepared with each batch of digestions. The detection limits were calculated by 11 determinations of the whole Program Blank Value. For precision, samples were analyzed using six replicates. The accuracy of the analyzed method was checked using certified reference material (GSW-07314) which is offshore marine sediment. The results for each heavy metal shown in Table 1 indicate a good agreement between the measured and assigned values. Only in the case of Pb was the measured value slightly lower than the assigned value. The relative standard deviations were lower than 6% for all metals.

### 2.4. Geoaccumulation Index

In order to better understand the current environmental status and metal contamination in the eastern Beibu Gulf, the geoaccumulation index (I_geo_) was calculated to assess the extent of sediment contamination in the study area. It was originally defined in 1979 [28] and can be calculated using the following expression:

(1) $$I_{\mathrm{geo}}{=\mathrm{Log}}_{2}[\frac{C_{n}}{1{.5*B}_{n}}],$$

where C**_n_** is the measured content of the examined metal “n” in the sediment; B**_n_** is the geochemical background content of the metal “n”, and factor 1.5 is the background matrix correction factor for lithogenic effects.

## 3. Results and Discussion

### 3.1. Granulometry and General Characteristics

**Figure 2:**
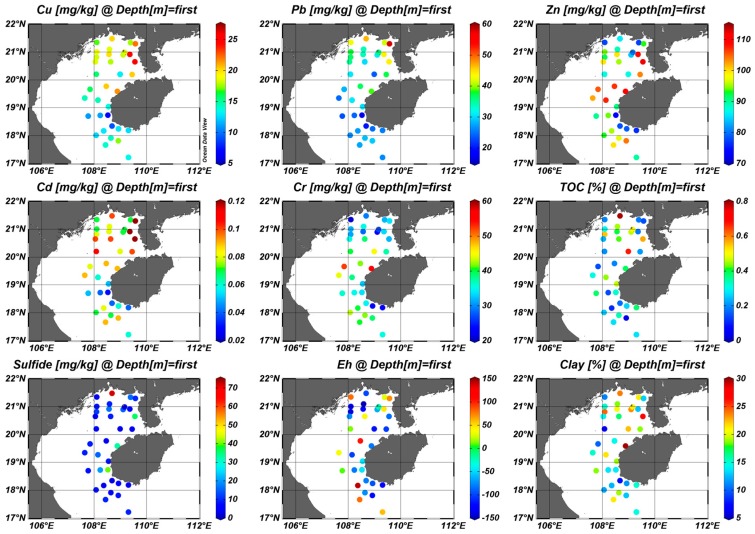
Distribution of heavy metals, total organic carbon (TOC), sulfide, Eh and clay in sediments from the eastern Beibu Gulf.

As shown in Figure 2, the contents of organic carbon and sulfide varied from 0.03~0.79% and 2.5~71.6 mg/kg, with the highest levels at station B21 which is located in the coastal zone of Guangxi Province. The results showed that the mean content of clay (grain size: 0.01–3.9 μm) for all sediment samples was 16.89%, with a range of 6.36% to 29.72%, and the values of the Eh ranged from −150 to 142. In the northernmost portion of study area, the weathering rock from surrounding land maybe influences the distribution of clay, organic carbon and sulfide [29]. The high concentrations of organic carbon and sulfide present in the coastal zone indicated that these are mainly terrigenous inputs, such as plant detritus from mangroves [3].

### 3.2. Concentrations of Heavy Metals in the Sediments

The concentrations of the five heavy metals in the surface sediments from the eastern Beibu Gulf varied from 6.72 to 25.95 mg/kg for Cu, 16.99 to 57.98 mg/kg for Pb, 73.15 to 112.25 mg/kg for Zn, 0.03 to 0.12 mg/kg for Cd and 20.69 to 56.47 mg/kg for Cr.

The results obtained from our study were compared with those from other regions in the world (Table 2). The average concentrations of Pb and Cd in the eastern Beibu Gulf were significantly higher, while the Zn concentration was lower, if we compared our data to that gained from the Guangxi province inshore investigated in 1998 [30]. The differences in concentration clearly showed that anthropogenic inputs of Pb and Cd have increased in the study area during the past 10 years. The mean values of selected metals were low compared to those from neighboring regions, such as the Pearl River Estuary [31], Pearl River [32] and Victoria Harbour [33], suggesting that heavy metal pollution is not a key issue in the eastern Beibu Gulf at present. In comparison with Taranto Gulf [34] and Thermaikos Gulf [35], the mean concentrations of Cu, Pb, Zn, Cd and Cr in the eastern Beibu Gulf were all lower. The concentrations of Cu, Zn, Cd and Cr were lower than in Puerto Rico [36], while the concentration of Pb was higher. Furthermore, comparing with Leizhou Bay, the concentrations of Cu, Cd and Cr were also lower, while the concentrations of Pb and Zn were higher [37,38].

The concentrations of metals in this study were compared with other recent research studying sediments (Table 2) [25,39], which indicated that the concentrations of most metals (Cu, Pb, Cd, Cr) were generally increasing from 2007 to 2013. Additionally, concentrations of Pb and Cd had obviously increased throughout the different sampling years (2007, 2011 and 2013), which reflects the influence of anthropogenic activities. Some studies have concluded that the metals risk in coastal Guangxi has increased in the past few years due to economic development and urbanization [39]. The eastern Beibu Gulf has been affected by industrial activities, such as petroleum production, printing industries and power plants [39], which increase the metals risk in sediments.

### 3.3. Assessment of Trace Metal Pollution

The metal concentrations were examined against the Marine Sediment Quality standard (GB 18668-2002), which was issued by the China State Bureau of Quality and Technical Supervision (CSBTS, 2002) (see Table 2). It has three standard criteria for marine sediments, among which the primary sediment standard criterion is the strictest and is applied to protect marine life habitats that include rare and endangered species, as well as places for human recreation and sports. Our results showed that the concentrations of heavy metals in the sediments from the study area met the primary standard criterion. Therefore, a general conclusion could be drawn that sediments in the eastern Beibu Gulf have not been significantly affected by metal pollution.

Since we did not have background values for metals in sediments from the Beibu Gulf, the I_geo_ was calculated using accepted earth crust values [40]. The earth crust values were in mg/kg: 45 for Cu, 20 for Pb, 95 for Zn, 0.3 for Cd, and 90 for Cr. According to the classification from Müller [41], I_geo_ < 0 is unpolluted, 0–1 is unpolluted to moderately polluted, 1–2 is moderately polluted, 2–3 is moderately to highly polluted, 3–4 is highly polluted, 4–5 is highly to very highly polluted, and >6 is very highly polluted.

The I_geo_ values deduced from the measured data in the study area are listed in Table 3. The I_geo_ values were −3.33~−1.38 for Cu, −0.82~0.95 for Pb, −0.96~−0.34 for Zn, −3.91~−1.91 for Cd and −2.71~−1.26 for Cr, respectively. The I_geo_ values of Cu, Zn, Cd and Cr in all stations were less than zero (I_geo_ < 0), indicating that the eastern Beibu Gulf had not been polluted by these metals. In contrast, most stations located in the northeast of the Beibu Gulf had a lower degree of Pb pollution (I_geo_ = 0~1). The Pb contamination in the coastal zone of Guangxi Province and northwest of Hainan Island might be caused by non-point sources [42,43,44].

### 3.4. Spatial Distribution Patterns

As shown in Figure 2, the distributions of Pb and Cd were similar to that of Cu, and significant correlations among Cu, Pb and Cd were also found (Table 4). The highest concentrations of Cu and Cd (25.95 mg/kg and 0.12 mg/kg, respectively) were found in site J07, which is located in the coastal area of Guangxi Province. The maximum Pb concentration at station B40 was 57.98 mg/kg. The levels of Cu, Pb and Cd in surface sediments from the northern Beibu Gulf were higher than those from the south. Alongside Pb, as a result, Cu and Cd may also be influenced by anthropogenic activities. The patches with higher concentrations of the above heavy metals were mainly confined to the coastal areas of Guangxi Province, Leizhou Peninsula and the northwest coast of Hainan Island (Figure 2). 

Metals from anthropogenic activities were mostly associated with bioavailable fractions. Additionally, according to a study about metals speciation in Beibu Gulf, Pb concentrations in bioavailable fractions were higher than in residual fractions, while Cu and Cd concentrations in exchangeable and carbonate fractions were relatively higher than other bioavailable fractions [44]. Therefore, we can draw the conclusion that the discharge of terrestrial pollutants was probably from the coastal areas of Guangxi Province or the Pearl River Estuary delivered via the Qiongzhou Strait. Meanwhile, there are some industrial activities around the coastal areas of Guangxi Province, such as coal-fired power plants, mineral mining and metallurgical industry, which can discharge waste into aquatic ecosystems [45]. A hotspot of Cd was identified at the center of Beibu Gulf, which was possibly affected by input from Guangxi province or Qiongzhou Strait.

Compared to the above three metals, Zn and Cr showed similar spatial distributions in the study area (Figure 2). The highest concentration of Cr in sediments was at station J41 (56.47 mg/kg), and the highest concentration for Zn was at station J07 (112.25 mg/kg). The high values of heavy metals around the northwest coast of Hainan Island and the Qiongzhou Strait might be related to the river inflow input from the coastal area of Hainan Island, and Qiongzhou Strait. Rivers in the northwest coast of Hainan Island can discharge metals into the Beibu Gulf. For example, Naidu River, the largest river in Hainan Island, discharged sediment detritus into Qiongzhou Strait. The Wenlan River, flowing into the northeast Beibu Gulf, contained high concentrations of metals from industrial sewage, which resulted in higher concentrations of metals at the northwest coast of Hainan Island than in the central Beibu Gulf [46]. Concentrations of Zn and Cr increased from the coast to the central areas in the south of the Gulf, which may be influenced by currents from the South China Sea [5]. 

However, according to the results of the I_geo_, the impacts of anthropogenic activities for increasing Zn and Cr were limited compared to natural bedrock. Considerably high concentrations of Cr were found in the residual fraction which was fairly stable and at a less bioavailable phase [44,47] and therefore indicated the influence of natural bedrock. The relatively high concentrations of Zn and Cr in Fe/Mn oxide fraction around the Qiongzhou Strait indicated that the diagenetic mobilization of metals and current through the Qiongzhou Strait may affect the distribution [44,48].

### 3.5. Cluster and Correlation Analyses

**Figure 3:**
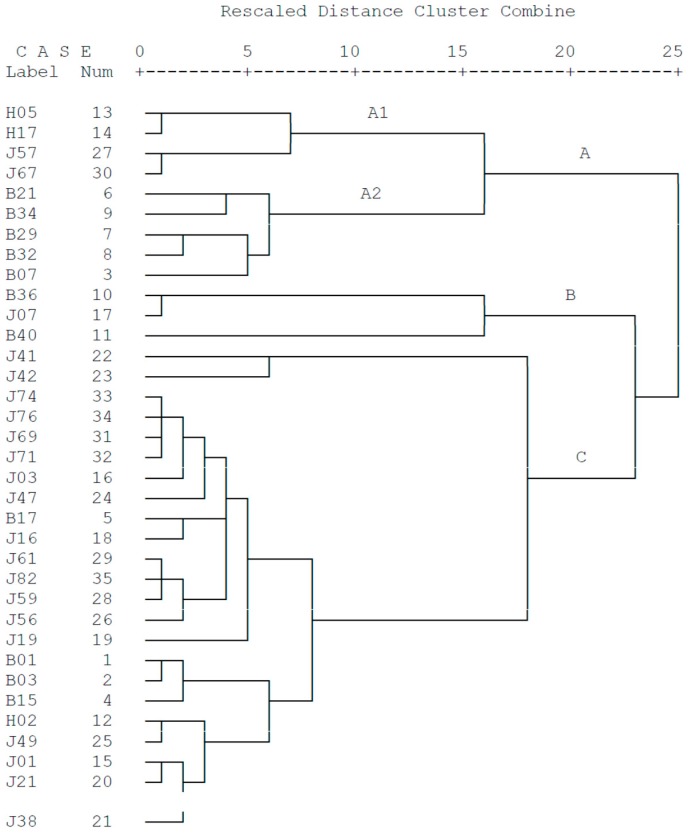
Cluster tree of the sampling stations in the eastern Beibu Gulf.

Hierarchical clustering analysis (HCA) was applied to evaluate the results obtained. The three groups (A, B and C) of sampling stations with different geographical distributions were clearly separated (Figure 3). Clusters A1 and A2 represented the stations off the southwest coast of Hainan Island and the coastal areas of Guangxi province, respectively. A2 may be related to local anthropogenic activities (i.e., discharge of industrial wastewater and municipal sewage, coal combustion and smelting) [45], while A1 was possibly associated with local river input from Hainan Island. Group B contained stations located in the coastal zone of the Leizhou Peninsula, and was represented by stations with higher levels of most metals in the sediments due to the inflow from Pearl River Estuary through the Qiongzhou Strait. All other stations belonged to Group C, which was influenced by various factors such as industry activities, river input or natural bedrock. Results of the HCA showed that sediment samples from different areas had different geochemical behavior and characteristics. 

Table 4 shows the correlation coefficients between heavy metals and some general characteristics of the surface sediments from the eastern Beibu Gulf. A significant positive correlation was found between Cu content and clay, which indicates that clay might be a carrier for Cu in the marine environment [31]. However, our results also showed that the interrelationships between metals and organic carbon, sulfide or Eh were not significant, indicating that most of the metals are not associated with organic carbon, sulfide or Eh. It was similar to results from Daya Bay [49]. The results showed that the correlation coefficients among Cu, Cd and Pb in the study area (r > 0.6) are significant, which suggested that similar processes governed the behavior of these metals [50]. Combined with the above discussion, these three metals may be from industrial activities. Zn and Cr also showed a significant relationship with the natural environment. Overall, our results indicated that the distribution of individual heavy metals in the surface sediments from Beibu Gulf was complex.

## 4. Conclusions

In this study, we found that although the concentrations of Cu, Pb, Zn, Cd and Cr in surface sediments were close to the primary standard criterion, the I_geo_ results showed that Pb pollution did exist in this area. Relatively high concentrations of Cu, Pb and Cd were found in the coastal areas of Guangxi Province, Leizhou Peninsula and off the northwest coast of Hainan Island, while high levels of Zn and Cr were observed off the northwest coast of Hainan Island. Spatial distribution patterns of these heavy metals show that bioavailable fractions of Pb were higher than those in the residual fractions, while Cu and Cd concentrations in the exchangeable and carbonate fractions were relatively higher than other bioavailable fractions. The diagenetic mobilization of metals and current through the Qiongzhou Strait may affect the distribution of metals. The result of HCA demonstrated three groups of stations. A significant positive correlation was observed between the content of Cu and clay, and significant correlation coefficients among Cu, Cd and Pb were also found in the study area, indicating that they were controlled by similar processes and had similar behavior. No significant correlations were found between the metals and organic carbon, sulfide or Eh, indicating that these latter factors play minor roles in binding the metals in the sediments.

## Figures and Tables

**Table 1:** Measured and certified values of metal concentrations in standard reference material.

Element	Method Blank (μg/L)	Limit of Detection (μg/L)	Relative Standard Deviation (%)	Measured Value (mg/kg)	Assigned Value (mg/kg)
Cu	1.23	0.04	1.6	27.00 ± 0.60	28 ± 2
Pb	5.47	0.21	2.6	16.40 ± 0.60	23 ± 4
Zn	7.00	1.41	1.2	80.10 ± 1.29	77 ± 6
Cd	0.017	0.009	5.3	0.21 ± 0.015	0.17 ± 0.04
Cr	2.60	0.08	0.9	46.2 ± 0.52	46 ± 8

**Table 2:** Comparison of the concentrations of heavy metals in the study area with values from the literature (unit: mg/kg).

Study Area	Cu	Pb	Zn	Cd	Cr	References
Eastern Beibu Gulf	16.37	32.23	93.12	0.08	35.51	This study
Beibu Gulf	-	36.22	-	0.12	-	[25]
Beibu Gulf	27.07	46.56	73.6	0.16	59.50	[39]
Coastal sea area, Guangxi Province, China	18.3	19	-	0.025	-	[30]
Pearl River Estuary, China	46.8	47.9	140	-	87.6	[31]
Pearl River, South China	348.0	102.6	383.4	1.72	93.1	[32]
Victoria Harbour	45.2–3789.5	47.4–138.1	97.9–610.4	2.61–3.33	57.5–601.2	[33]
Taranto Gulf	47.4	57.8	102.3	-	85.9	[34]
Puerto Rico	106	11	-	0.13	55	[35]
Leizhou Bay	4.51–30.5	8.94–32.2	-	0.18–0.67	-	[37]
	22	21.9	60.4	0.12	60	[38]
Thermaikos Gulf	80	77	184	-	47	[35]
Primary standard criteria	35.0	60.0	150.0	0.5	80	a

“-”: not available; “a”: values from national standard GB 18668-2002 (CSBTS, 2002).

**Table 3:** Geoaccumulation index (I_geo_) values of heavy metals in the eastern Beibu Gulf.

Station	Cu	Pb	Zn	Cd	Cr
B01	−1.89	0.28	−0.63	−2.49	−2.12
B03	−1.84	0.33	−0.60	−2.68	−2.11
B07	−2.00	0.51	−0.88	−2.68	−2.71
B15	−1.76	0.17	−0.52	−2.49	−2.25
B17	−1.87	0.18	−0.68	−2.49	−1.93
B21	−1.74	0.63	−0.73	−2.17	−2.21
B29	−1.91	0.04	−0.76	−2.68	−2.57
B32	−1.88	0.26	−0.83	−2.68	−2.34
B34	−1.94	0.46	−0.89	−2.68	−2.09
B36	−1.43	0.55	−0.39	−1.91	−2.02
B40	−1.61	0.95	−0.61	−1.91	−2.01
H02	−1.96	−0.04	−0.45	−2.32	−1.79
H05	−2.40	−0.30	−0.86	−2.91	−2.57
H17	−2.42	−0.24	−0.90	−3.49	−2.54
J01	−1.87	0.24	−0.52	−2.17	−1.93
J03	−1.93	0.00	−0.58	−2.17	−1.79
J07	−1.38	0.55	−0.34	−1.91	−2.09
J16	−2.11	0.03	−0.71	−2.03	−1.76
J19	−2.37	−0.34	−0.71	−2.49	−1.58
J21	−1.73	0.32	−0.46	−2.17	−1.85
J38	−1.73	0.29	−0.42	−2.32	−1.70
J41	−1.65	0.36	−0.36	−2.32	−1.26
J42	−2.09	−0.09	−0.44	−2.49	−1.36
J47	−2.22	−0.30	−0.48	−2.32	−1.61
J49	−2.21	0.04	−0.41	−2.91	−1.82
J56	−2.30	0.01	−0.64	−3.17	−2.06
J57	−3.33	−0.82	−0.96	−3.91	−2.01
J59	−2.60	−0.32	−0.60	−3.49	−1.94
J61	−2.62	−0.38	−0.66	−3.17	−1.90
J67	−2.89	−0.60	−0.87	−3.49	−1.96
J69	−2.40	−0.16	−0.58	−2.49	−1.75
J71	−2.45	−0.21	−0.61	−2.68	−1.66
J74	−2.23	−0.07	−0.55	−2.68	−1.69
J76	−2.25	−0.13	−0.52	−2.32	−1.74
J82	−2.34	−0.21	−0.67	−2.91	−1.93

**Table 4:** Pearson correlation matrix for the metal concentrations and sediment properties.

	Cu	Pb	Zn	Cd	Cr	OC	Sulfide	Eh	Clay
Cu	1								
Pb	0.859 **	1							
Zn	0.539 **	0.258	1						
Cd	0.812 **	0.681 **	0.579 **	1					
Cr	−0.038	−0.191	0.589 **	0.236	1				
OC	0.153	0.096	−0.111	0.013	−0.173	1			
Sulfide	0.173	0.243	0.009	0.080	−0.037	0.503 **	1		
Eh	−0.013	0.119	0.031	0.218	0.137	−0.441 *	−0.230	1	
Clay	0.390 *	0.278	0.234	0.140	0.030	0.722 **	0.465 **	−0.334	1

** Correlation is significant at the 0.01 level (2-tailed). * Correlation is significant at the 0.05 level (2-tailed).
